# Supplementary material for: Manufacturing DNA in E. coli yields higher-fidelity DNA than in vitro enzymatic synthesis
Source: Mol Ther Methods Clin Dev. 2024 Feb 28;32(2):101227. doi: 10.1016/j.omtm.2024.101227 (PMC10951457; doi:10.1016/j.omtm.2024.101227)
Supplement: Document S1. Figure S1 [file mmc1.pdf]

OMTM, Volume *32*

Supplemental information

Manufacturing DNA in *E. coli* yields higher-fidelity DNA than *in vitro* enzymatic synthesis

Steven J. Hersch, Siddarth Chandrasekaran, Jamie Lam, Nafiseh Nafissi, and Roderick A. Slavcev

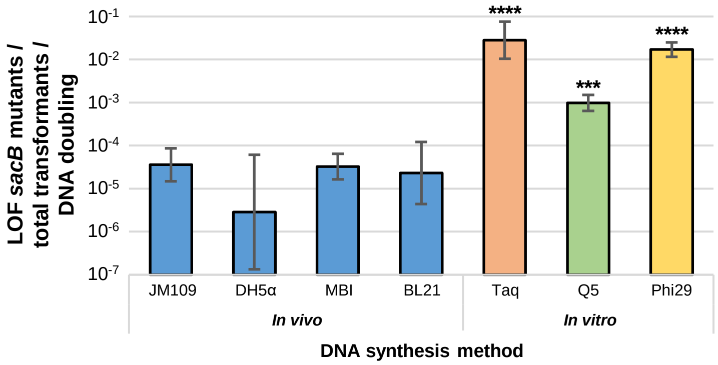

Figure S1: LOF mutation rates identified using the SuTox method with a *SacB-cat* cassette that does *not* contain ITRs. Data is shown as LOF mutant colonies / total transformant colonies / number of DNA doublings. DNA was synthesized *in vivo* (in four different *E. coli* strains) or *in vitro* (PCR with Taq or Q5 polymerases, or RCA with Phi29 polymerase). Bars show the average of at least three biological replicates and error bars show one standard deviation. One-way ANOVA with Dunnett's test (compared to DH5α): ***, $p < 0.001$; ****, $p < 0.0001$.
